# Editorial: The Complex Interaction Between Biological, Metabolic and Neurologic Dysregulation in Obstructive Sleep Apnea

**DOI:** 10.3389/fpsyt.2021.770930

**Published:** 2021-10-05

**Authors:** Georgia Trakada, Carolina Lombardi, Beat Knechtle

**Affiliations:** ^1^School of Health Sciences, National and Kapodistrian University of Athens, Athens, Greece; ^2^Istituto Auxologico Italiano (IRCCS), Milan, Italy; ^3^Institut für Hausarztmedizin, Universitätsklinikum Zürich, Zurich, Switzerland

**Keywords:** obstructive sleep apnea (OSA), visceral obesity, cardiometabolic risk, chronic mental disease, inflammation

Obstructive sleep apnea (OSA) is a prevalent chronic disorder characterized by repetitive apneas and hypopneas during sleep, leading to intermittent hypoxia (IH) and recurrent arousals from sleep, leading to sleep fragmentation and reduced sleep efficiency (1). Accumulated evidence suggests that OSA—and the underlying biological, metabolic, and neurologic dysregulation—is closely associated with cardiometabolic disorders, neurocognitive dysfunction, and chronic mental diseases (2–4) (Figure 1). In this Research Topic we aimed to further evaluate symptoms and specific characteristics of OSA patients and their possible interactions in the progression to different disease states in each individual.

**Figure 1:**
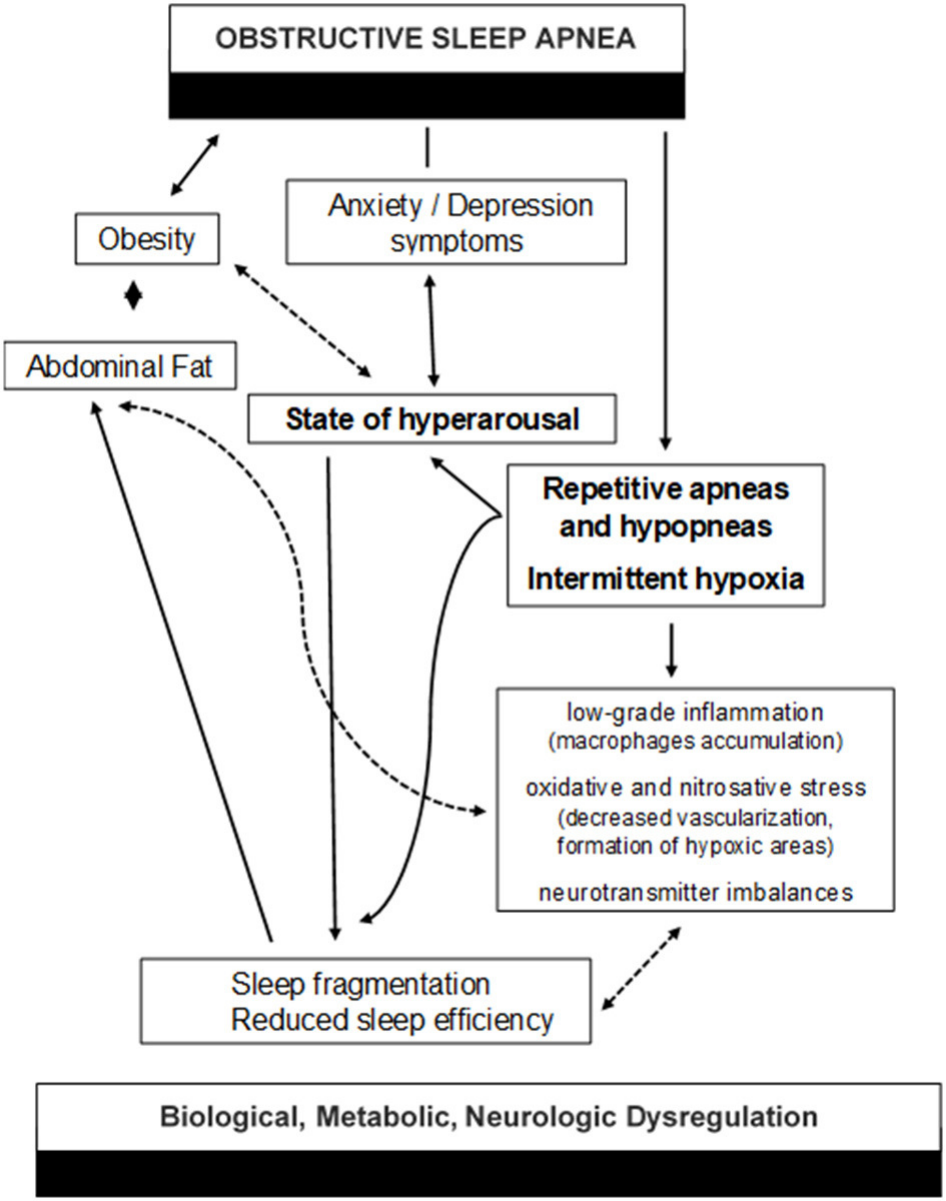
The vicious cycle between biological, metabolic, and neurologic dysregulation in OSA.

In a nationwide, stratified, epidemiological survey in Cyprus (*n* = 4,118), Frangopoulos et al. demonstrated that 155/264 randomly selected adults that underwent a type III sleep study, had an Apnea/Hypopnea Index (AHI) ≥ 5. These newly diagnosed OSA patients were categorized as symptomatic or asymptomatic, according to the Epworth Sleepiness Scale (ESS) and/or the Athens Insomnia Scale (AIS). The two groups did not differ in terms of OSA severity and in cardiometabolic comorbidities; however, symptomatic patients expressed more often both depression and anxiety when compared to asymptomatic patients, because of a poorer subjective sleep quality.

In another cross-sectional study of working adults in Kuwait (*n* = 651), Al-Qattan et al. estimated that 20% of participants were at high risk for OSA, according to the Berlin Questionnaire. The risk factors associated with elevated OSA probability were older age, obesity, lower educational level, current smoking status, no physical activity, increased television viewing, increased working hours, and short sleep duration. Individuals at high risk for OSA suffered more often than those at low risk from arterial hypertension, diabetes mellitus, insomnia, and depression.

Mohammadi et al. revealed that moderate/severe OSA was associated with a significantly lower spindle density in N3 and a shorter spindle duration in N2, whereas mild OSA showed no significant adverse effect on sleep spindle characteristics. Sleep spindles are associated with cognitive, emotional, and social processes and lower sleep spindle activity can be responsible for the deterioration of memory and cognition in OSA.

Feng et al. evaluated the associations between objective sleep parameters derived via polysomnography (PSG) and metabolic indices. Although the observed associations were weak, both sleep structure and sleep duration affected the metabolic status of the participants. Specifically, the microarousal index (MAI)—an indicator of sleep fragmentation—significantly correlated with glucose levels and lipids metabolism, independently of respiratory indices during sleep.

Finally, Qin et al. reviewed the data about heart rate variability (HRV), as a reliable and non-invasive measure of neuro-cardiac autonomic regulatory mechanisms in different aspects of OSA. Altered HRV features can be good predictors of cardiovascular morbidity and mortality in OSA.

The major challenge in studying OSA is that the disease is heterogeneous and multifactorial. The clinical OSA phenotypes can vary in symptoms; sleepy vs. insomniac vs. asymptomatic patient. Also, the underlying pathophysiological phenotypes of OSA can be anatomical or non-anatomical, like functional impairment of pharyngeal dilator muscles during sleep, low respiratory arousal threshold and increased propensity for awakenings, or respiratory control instability because of the high loop gain mechanism. However, behind OSA and apnea-related parameters (AHI, average saturation of hemoglobin with oxygen as measured by pulse oximetry [SpO_2_], and percentage of sleep time with SpO_2_ < 90%, t < 90), there is always a common denominator, the sleep process, that affects clinical picture and comorbidity. In all valuable contributions in this Research Topic, subjective or objective disturbed sleep was mainly associated with symptoms of anxiety and depression, neurocognitive defects, and cardiometabolic dysregulation.

The vicious cycle between biological, metabolic, and neurologic dysregulation in OSA still not clear and more studies are needed to be conducted to better ascertain these relationships. Future studies should extensively focus on sleep, in terms of quality, duration, circadian rhythms and architecture—both in OSA patients and in general population—to better clarify these complicated underlying pathophysiological mechanisms.

## Author Contributions

All authors listed have made a substantial, direct and intellectual contribution to the work, and approved it for publication.

## Conflict of Interest

The authors declare that the research was conducted in the absence of any commercial or financial relationships that could be construed as a potential conflict of interest.

## Publisher's Note

All claims expressed in this article are solely those of the authors and do not necessarily represent those of their affiliated organizations, or those of the publisher, the editors and the reviewers. Any product that may be evaluated in this article, or claim that may be made by its manufacturer, is not guaranteed or endorsed by the publisher.

## References

[1] American Academy of Sleep Medicine. International Classification of Sleep Disorders: Diagnostic and Coding Manual. 3rd ed. Westchester, NY: American Academy of Sleep Medicine (2014).

[2] Kallianos A Trakada G Papaioannou T Nikolopouloss I Mitrakou A Manios E. Glucose and arterial blood pressure variability in obstructive sleep apnea syndrome. Eur Rev Med Pharmacol Sci. (2013) 17:1932–7. 23877859

[3] Trakada G Chrousos G Pejovic S Vgontzas A. Sleep apnea and its association with the stress system, inflammation, insulin resistance and visceral obesity. Sleep Med Clin. (2007) 2:251–61. 10.1016/j.jsmc.2007.04.003 18516220 PMC2128620

[4] Knechtle B Economou NT Nikolaidis PT Velentza L Kallianos A Steiropoulos P. Clinical characteristics of obstructive sleep apnea in psychiatric disease. J Clin Med. (2019) 8:534. 10.3390/jcm8040534 31003451 PMC6518048

